# Initiatives Toward Clinical Boron Neutron Capture Therapy in Japan

**DOI:** 10.1089/cbr.2022.0056

**Published:** 2023-04-05

**Authors:** Akira Matsumura, Tomoyuki Asano, Katsumi Hirose, Hiroshi Igaki, Shinji Kawabata, Hiroaki Kumada

**Affiliations:** ^1^ Ibaraki Prefectural University of Health Sciences, Ibaraki, Japan.; ^2^ Proton Medical Research Center, University of Tsukuba, Ibaraki, Japan.; ^3^ Stella Pharma Co. Ltd., Osaka, Japan.; ^4^ Department of Radiation Oncology, Southern Tohoku Hospital, Fukushima, Japan.; ^5^ Division of Boron Neutron Capture Therapy Medical Research, Exploratory Oncology Research & Clinical Trial Center, National Cancer Center, Tokyo, Japan.; ^6^ Department of Neurosurgery, Osaka Medical and Pharmaceutical University, Osaka, Japan.

**Keywords:** BNCT, clinical boron neutron capture therapy, Japan

## Abstract

Boron neutron capture therapy (BNCT) has been performed at nuclear research reactors for many years. The development of accelerators for BNCT resulted in a paradigm shift from research to real clinical applications. In Japan, BNCT was approved as a clinical therapy covered by the National Health Insurance in 2020. In this article, the status of BNCT in Japan is briefly introduced.

## Boron Neutron Capture Therapy Facilities in Japan

There are currently five institutions for clinical boron neutron capture therapy (BNCT) in Japan.

### Southern Tohoku hospital

Cyclotron-based BNCT equipment with a beryllium target (NeuCure^®^, 30 MeV, 1 mA; Sumitomo Heavy Industries Ltd.) was installed,^1^ and a clinical trial for recurrent head and neck cancers^2^ and recurrent malignant glioma (MG) was conducted. Based on the approval of this clinical trial, this hospital also performs medical treatments covered by the National Health Insurance for locally advanced or locally recurrent head and neck cancers using the boronated compound, Borofalan (^10^B) (Steboronin^®^; Stella Pharma Corporation).

### Osaka Medical and Pharmaceutical University (Kansai BNCT Medical Center)^3^

This facility installed a cyclotron-based BNCT equipment (NeuCure; Sumitomo Heavy Industries Ltd.), as well as performing clinical trials for head and neck cancers,^2^ recurrent MG,^4^ and recurrent high-grade meningioma^5^ using the accelerator with boronated compound, Borofalan (^10^B) (Steboronin^®^). Some of these projects are currently still underway. Medical treatments covered by the National Health Insurance for locally advanced or recurrent head and neck cancers are also performed in this facility.

### National Cancer Center Hospital^6^

Under a contract of joint research with Cancer Intelligence Care Systems (CICS), Inc., an accelerator-based neutron irradiation system, CICS-1, was developed, which is the world's first neutron irradiation system that adopts a solid lithium target as the neutron generation material. Because of this target material selection, CICS-1 has a compact beam-shaping assembly with a neutron beam moderator and a 24 cm thick magnesium fluoride (MgF_2_), which can be installed in a compact radiotherapy room within the hospital. Another prominent characteristic of this system is its vertical beam port. This is a unique aspect of BNCT systems, and the techniques and tools developed for photon/particle radiotherapy systems can be applied to BNCT.

CICS-1 was installed in the Department of Radiation Oncology of the hospital within the treatment area of ordinary patients, which is also a distinctive feature of a BNCT system. All members of the medical staff, including physicians, medical physicists, radiation technologists, and nurses, participated cross-functionally between BNCT and photon radiotherapy services.

The CICS-1 has a radio-frequency quadrupole (RFQ) linear accelerator system that accelerates protons to 2.5 MeV. The accelerator is 8 m long, and the internal dimensions of the accelerator room are 20 × 8 × 5 m. CICS-1 runs stably at a proton current of 12 mA. Equipment related to the power supply, cooling system, and maintenance service device was also installed in the accelerator room.

The treatment room is 8 × 7 × 3.5 m in size and is equipped with the Li target system, beam shaping assembly of the vertical beam port, a treatment couch with a 5-axis shift, and a patient monitoring system. Using this CICS-1 system, a maximum thermal neutron flux of 1.41 × 10^9^/cm^2^/s is available, and neutron irradiation can be completed within 1 h. The same neutron irradiation system of CICS-2 was installed in Edogawa Hospital, where the system was in preparation for the neutron beam for the start of the clinical trial.

### University of Tsukuba Hospital^7^

The University of Tsukuba, in collaboration with the High Energy Accelerator Research Organization (KEK), developed an RFQ+DTL-based accelerator (8 MeV and 2.1 mA) with a beryllium target (iBNCt Co. Ltd. with Toyama Co. Ltd. & iAC Co. Ltd.) was installed at the Ibaraki Neutron Medical Research Center in Tokai village. This facility has started conducting clinical trials for initially diagnosed MGs.

### Shonan Kamakura Hospital

This hospital constructed a particle therapy center and installed a proton therapy system and installed a BNCT accelerator (NuBeam^®^) developed by Neutron Therapeutics Co. Ltd. in 2021.

### Other preclinical facilities

There is a research-based accelerator project at Nagoya University that uses an electrostatic Dynamitron Accelerator (max. 2.8 MeV, 15 mA; IBA Co. Ltd. and Yagami Co. Ltd.).^8^

Another unique project is ongoing at Kyoto Prefectural University using silicon carbide (SiC)-based gantry-type accelerator installed at the “ROHM BNCT Center” on their campus.

Osaka University is planning to construct BNCT accelerators in the near future.

## BNCT as Insurance Medical Treatment

A clinical trial for recurrent head and neck cancer was initiated in 2014 for phase I using C-BENS (NeuCure) and Borofalan (^10^B) (Steboronin^®^). From 2016, a phase II trial was initiated. A single dose of Borofalan (^10^B) was administered intravenously at a rate of 200 mg/kg/h during the first 2 h and then at 100 mg/kg/h during neutron beam irradiation. Single-dose neutron beam irradiation was initiated 2 h after the start of Borofalan (^10^B) administration. Further details are provided in this journal.

This clinical trial was approved by the Pharmaceuticals and Medical Devices Agency (PMDA; Japanese FDA) in 2020, which was certified as a “pioneering medical product” and registered in the “SAKIGAKE designation system. The fast track review”,^9^ quickly obtained marketing approval from the central medical council (Ministry of Health Labor and Welfare; MHLW) in March 2020. Soon after, in June 2020, BNCT for locally advanced or locally recurrent head and neck cancer became a medical therapy covered by Japan's national health insurance.

The irradiation fee is about 2.4 million Japanese Yen (JY) and the cost of boron drug, which depends on the weight of the patient, is around 1.7 million JY (in total ca. 4.1 M JY). In Japan, if the total amount exceeds the cost-sharing maximum amount, then the excess amount is paid by the Health Insurance Association as “total high-cost medical care benefits,” depending on the income level of the patient (the limit of payment varies roughly from 40,000 JY up to 380,000 JY in the case of BNCT).^10^

## Current Clinical Indications and Ongoing Clinical Trials for BNCT

### Clinical trial for recurrent head and neck cancers

In Japan, BNCT is currently available under the national health insurance system for patients with recurrent or locally advanced head and neck cancers. To obtain the approval of the Japanese government, preclinical studies were conducted first. Thereafter, a phase I study was initiated in 2012 to evaluate the safety of this treatment. Next, a phase II study was conducted in 2016 to confirm its efficacy and safety. Based on these results, the neutron irradiation system and boron agent, Borofalan (^10^B), were approved by the PMDA in 2020 and are currently being used in clinical practice.

The JHN001 study was conducted by Kawasaki Medical College and Kyoto University Research Reactor. This consisted of a phase I trial with a dose escalation study. The subjects were patients with unresectable recurrent squamous cell carcinoma (SCC) and recurrent and locally advanced non-SCC (n-SCC). In cohort 1, six patients were enrolled and treated with a prescribed dose of 10 Gy-Eq as the maximum mucosal dose. In cohort 2, three patients were enrolled and treated with a prescribed dose of 12 Gy-Eq. The prescription of 12 Gy-Eq was derived from the reported dose for the endpoint of lethal oral mucosal ulcer formation in rats. Patients were administered ^10^B for 2 h, and the concentration of ^10^B was measured using blood samples.

Immediately after these measurements, neutron irradiation was performed with a continuous infusion of ^10^B until the maximum mucosal dose reached the prescribed dose. The tumor dose was passively administered. In cohort 1, one person developed Gr3 dysphagia, however, this was primarily due to the exacerbation of the tumor. BNCT is considered to have an indirect effect. In both cohorts, no issues were encountered and patient safety was ensured.

The JHN002 study was conducted to confirm the efficacy and safety of the Southern Tohoku BNCT Research Center and National Cancer Center Hospital. A total of 21 patients were enrolled, all of whom had unresectable recurrent SSC, or recurrent or locally advanced SSC. The primary endpoint was response rate within 90 d of treatment. The secondary endpoints included efficacy and safety evaluations. Eight patients with SCC and 13 patients with non-SCC were enrolled in the study. All patients had a history of irradiation, especially in case of SCC. Regarding efficacy, SCC had a complete response (CR) of 50% and an overall response rate (ORR) of 75%. The ORR for all the patients was 71%. Patients with SCC had a 2-year overall survival (OS) of 55% and a progression-free survival (PFS) of ∼1 year.

Despite this favorable response, toxicity was very low, with three patients having symptomatic grade 3 or higher adverse events, one each with grade 3 dermatitis, grade 3 mucositis, and grade 3 brain abscess. Comparing these results to those of other clinical trials on reirradiation for locally recurrent head and neck cancer and the EXTREME trial, the CR and ORR of JHN002 were comparable or better and were considered to be extremely safe.^2^

Currently, BNCT is available through the public health insurance system in Japan. This treatment is indicated for locally advanced or recurrent head and neck cancers. It also includes in-field secondary cancers after radiation therapy (RT). However, for patients who are not receiving standard treatment, including radiotherapy, standard treatment must be administered before BNCT.

To date, more than 100 patients were treated with BNCT under national health insurance system in Southern Tohoku BNCT Research Center. As a preliminary evaluation, the actual clinical results were investigated. The method was to analyze the treatment results of patients who underwent BNCT from June 2020 to February 2021. Patients with local recurrence and secondary cancer in the radiation field were included in the analysis. For the patient background, the treated sites included hypopharynx, oral cavity, parapharyngeal space, external auditory canal, level II lymph nodes, and so on. The median primary size was almost 10 mL. For safety, Grade 3 adverse events including stomatitis, vomiting, decreased appetite, and mucositis, were observed in 30% of total patients. Late effects included prolonged stomatitis. Hair loss was observed in 80%. Next, for efficacy, the CR rate and the response rate were almost same as the result of the JHN002 study. Compared to other clinical trials of reirradiation + chemotherapy for recurrent head and neck cancer, the results of this analysis were quite favorable although preliminarily unofficial analyses. In summary, in Japan, the efficacy and safety of BNCT for recurrent and locally advanced head and neck cancer (R/LA-HNC) were confirmed in a phase II clinical trial (JHN002 Study). This treatment is now available under public health insurance system. As for the actual clinical data, preliminary analysis has confirmed the treatment efficacy is similar to that of JHN002.

### Japanese clinical trials of accelerator-based BNCT for recurrent MG^4^

The aim of this study was to assess the safety and efficacy of accelerator-based BNCT (AB-BNCT) using a cyclotron-based neutron generator, BNCT30 (NeuCure, Sumitomo Heavy Industries Ltd., Tokyo, Japan), and ^10^B-boronophenylalanine, SPM-011 Borofalan (^10^B) (Steboronin^®^; Stella Pharma Corporation, Osaka, Japan), in patients with recurrent MGs, chiefly glioblastoma (GB). A multi-institutional open-label, phase II clinical trial (JG002) for 27 recurrent cases of MG, including 24 GB, was conducted using this AB-BNCT system and 500 mg/kg of SPM-011. The primary endpoint was the 1-year survival rate, while the secondary endpoints were median overall survival (mOS) and median progression-free survival (mPFS). The 1-year survival rate and mOS of recurrent GB cases were 79.2% (95% CI: 57.0–90.8) and 18.7 months (95% CI: 12.9-NE), respectively, and the median PFS was 0.9 months in the image-based evaluation criteria using RANO.

The most prominent adverse event reported in the JG002 trial was brain edema. Based on the results of this clinical trial, the following conclusions were drawn. AB-BNCT is useful for MG patients after surgery or radiotherapy, and it should be introduced into clinical practice as a treatment option as soon as possible. Currently, this therapy is limited to facilities where the appropriate device is installed; however, unlike nuclear reactors, it is a medical device that can be installed immediately within most facilities. Therefore, in the near future, the global expansion of the AB-BNCT system and validation through large-scale international joint trials are expected.

Recurrent MG is a life-threatening disease with poor prognosis. Currently, there are no satisfactory life-prolonging treatments available. Because the 1-year survival rate for recurrent MG is inadequate, the primary endpoint of the clinical trial was discussed using the 1-year survival rate, with local control measures, including response rate, disease control rate, and PFS, as the secondary endpoints. Large randomized, double-blind controlled trials of AVAglio and RTOG0825 in primary GB using bevacizumab (Bev) showed prolonged PFS, but not OS.^11,12^ This suggests that although radio graphical improvement is an important factor and has the potential to improve patient performance, it does not contribute to the prolongation of survival in MG.

The results of AB-BNCT suggest that the OS outcomes of patients undergoing BNCT, as well as the 1-year survival status as the primary endpoint, were greatly improved. Although the secondary endpoints of PFS and response rate based on RANO criteria were clearly poor (PFS: 0.9 month, 95% CI: 0.8–1.0 and response rate: 0%), there was a significant improvement in the life expectancy, highlighting the difficulty of evaluating this therapy, especially for central nervous system (CNS) tumors, using existing imaging methods, as will be discussed later. Such a problem has often been pointed out, and has also become a problem in angiogenesis-inhibiting therapy and recent immunotherapy for CNS tumors. In turn, this has led to proposals for an optimized evaluation method as an alternative to the previous image evaluation criteria that have been commonly used for other solid cancers.

The large discrepancy between the OS and PFS in this study suggests that there is an antitumor effect that is difficult to evaluate using the commonly used image evaluation criteria in clinical trials, such as RECIST and RANO, to assess tumor reduction/enlargement in MG. Because of the difficulties associated with evaluation using radiographical endpoints, this BNCT trial reported a much shorter PFS than OS; however, the patient's Karnofsky Performance Scale (KPS) did not decline after the decision of progression by imaging study. As shown in the present study, sufficient attention and assessment should be taken to prevent the occurrence or worsening of peritumoral edema.

MGs develop in an infiltrative manner, with no clear borders. Once the tumor grows and increases in activity, it develops fragile tumor vessels without the blood–brain barrier that can be visualized as a contrast-enhanced lesion. Therefore, it is difficult to distinguish the margins of the tumor without enhancing on contrast-enhanced imaging. Therefore, when conventional radiotherapy or stereotactic irradiation is used, the gross tumor volume is a clearly contrasted area on imaging that can be defined as a tumor mass, and there is much discussion on the clinical target volume. The targeting technique of BNCT is based on the distribution of drugs with biological targets and the distribution of neutron fluxes, which are targeted with or without contrast.

Early enlargement of the contrasted lesion was observed after BNCT, which was confirmed as progressive disease (PD); however, the newly contrasted area was the biologically targeted area, and this lesion was considered to indicate a treatment-affected tumor. Although there is a possibility that normal brain tissue may be affected, so-called brain radionecrosis, this possibility was refuted due to the fact that the new contrast-enhanced area was in close proximity to the original contrasted lesion in all cases, which is independent of neutron intensity and irradiation field, and does not cause isolated contrast-enhanced lesions to appear in remote areas (new lesions).

**FIG. 1.**
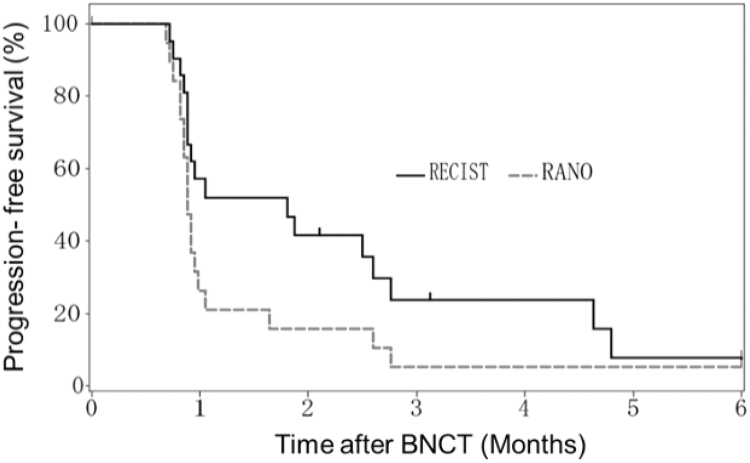
PFS evaluated using the RANO and RESIST criteria in patients with glioblastoma in accelerator-based BNCT trial (JG002). The 6-month PFS rate and mPFS of patients with recurrent GB were 7.9% (95% CI: 0.6–28.7) and 1.8 months (95% CI: 0.9–2.8) using the RECIST criteria, 5.3% (95% CI: 0.4–21.4) and 0.9 months (95% CI: 0.8–1.0) using the RANO criteria, respectively, by externally separated central review. BNCT, boron neutron capture therapy; PFS, progression-free survival; mPFS, median progression-free survival.

In the present study, a significant difference was observed in OS and PFS. However, at the same time, differences in the evaluation of PFS by RANO and RECIST were also observed (Fig. 1). RANO was designed to objectively assess noncontrast tumor aggravation in response to treatments with an antiangiogenic effect. The difference between the two assessments identified in this study was represented by the appearance or increase in cerebral edema with the appearance of the contrast area. Nevertheless, the time course of these patients after BNCT showed that KPS was stable even after a graphical PD assessment was performed.

Although early increases in the contrast area are a characteristic feature and a result of BNCT for the CNS, the disadvantage of this treatment effect is that it is associated with cerebral edema, as mentioned in its adverse events (AE), and antiedema treatment should be considered after BNCT for the CNS. Antiangiogenic agents have a significant therapeutic effect on the improvement of vasogenic cerebral edema associated with contrast lesions. In the case of primary brain tumors, the number of cases in which the contrast area shrinks with therapeutic intervention against the tumor itself is rare. Thus, there is a need to explore different treatment methods to determine the effectiveness of treatment, such as BNCT.

“Reirradiation” implies irradiating the same area again, including the same risk organs of the body that have already received RT. Reports in a variety of organs have shown that reirradiation is nearly as effective as the first dose for tumors. However, the problem with reirradiation is the tolerated dose to normal tissue around the tumor and the risk of developing serious AEs. Normal tissue around a tumor can safely receive only a limited amount of radiation, known as the “tolerable dose.” Stereotactic irradiation, IMRT, and brachytherapy have been used to avoid severe AEs caused by reirradiation; BNCT is also an example. Because the principle of biologically cell-selective cytotoxicity of BNCT is clearly different from that of these irradiations that physically target the tumor based on the image, it is expected to be effective in treating tumors that have been difficult to treat safely even using these reirradiation techniques.

Stereotactic radiotherapy has a median OS of less than a year, with limitations based on tumor size.^13^ In contrast, the results of the present study highlight the efficacy of reirradiation with BNCT in patients who relapse after radiotherapy. Safety analysis of all 27 patients with recurrent MG who underwent BNCT showed acceptable toxicity, and AEs did not represent a major safety hazard. This study demonstrated the efficacy and safety of “reirradiation” with BNCT, without exceeding the risks of conventional RT for MG.

Although local control is required for radiotherapy, including BNCT, prolonging the OS of MG patients, especially in recurrent cases, is the most promising treatment. The OS and PFS have been used in various clinical trials of MG, and the correlation between 1-year survival and 6-month PFS has been discussed previously.^14^ On the contrary, since the advent of Bev, the AVAglio^12^ and RTOG0825^11^ studies have shown the prolongation of PFS but not OS for MG. Thus, the relevant literature was evaluated to investigate the validity of establishing an efficacy assessment indicator for recurrent MG. Publications for randomized controlled trials with control groups in the literature since the AVAglio trial have reported a correlation between PFS and OS.^15–21^ This consideration supports previous publications describing the correlation between the 6-month PFS rates and 1-year survival.

However, four of these publications, which included only recurrent cases, were reviewed and no correlation was found.^15,17,19,21^ The results of these studies suggest that PFS may be an inadequate alternative measure of the OS for recurrent MG. There is no alternative measure to determine the effectiveness of a new type of treatment modality other than to directly evaluate OS in patients with recurrent GB.

The results of the JG002 study showed that BNCT using BNCT30 was safe and effective. Although there are difficulties in radiographical assessment that may be particular to BNCT for MG with a highly invasive nature, and the results were obtained in a small number of phase II patients, the OS of patients with recurrent MG with prior radiation undergoing BNCT was improved, even after considering the background of the patients who participated in the study. In terms of safety, although some adverse events were reported, these events were similar or less than those observed with other previously developed radiation modalities and were tolerable, considering that these were all reirradiation cases after radiotherapy. In conclusion, this phase II study demonstrates the clinical benefits of BNCT in patients with recurrent MG.

Several discussion points specific to BNCT are described in the following:

#### There is urgent need to develop optimal imaging evaluation criteria for BNCT, particularly for brain tumors

The short PFS on image-based criteria showed in the trial was a pseudo-progressed disease, changing the nonenhanced tumor to enhanced, mostly with brain edema. Therefore, biomarkers, especially imaging biomarkers to determine the long OS, are required. Boron distribution imaging (^18^FBPA positron emission tomography [PET]) would play a role in demonstrating this, but only prolonged OS is the true effect.

#### In BNCT, how is the dose prescribed?

Skin dose was used to consider the possibility of applying it to a wider area of whole-body cancer for future BNCT. Therefore, the biological effects on underlying normal organs at risk corresponding to the skin must be investigated in detail. It is hoped that the dose can be prescribed for the tumor (cancer), wherein ^18^FBPA PET scans represent a major breakthrough.

#### Is the dosing protocol for BPA optimal?

In this study, a protocol was adopted to ensure that the blood boron concentration before and after irradiation remained constant due to the fact that the prescribed dose should not depend on postevaluation. Stella Pharma's Borofalan (^10^B), Steboronin, was found to have a good safety profile in human trials and can be used in clinical trials without any severe problems. It is an essential drug for BNCT treatment.

#### What are the specific features of the accelerator neutron generation (Sumitomo) system used?

Currently, only fixed horizontal beams are available. Therefore, it is necessary to move patients to obtain an optimal position. A unique feature of the Sumitomo device, NeuCure, is that it has both a chair and a couch system to allow the patient to adopt an optimal beam angle and treatment position to target the treatment site. The Sumitomo system has a separate setup room away from the treatment room that is connected by a rail delivery system. This ensures that the patient can be automatically transferred to the treatment room, and is a patient- and hospital staff-friendly system.

### Other ongoing clinical trials in Japan

#### Skin melanoma and angiosarcoma (JapicCTI-195062)^22,23^

This phase I clinical trial is currently performed at the National Cancer Center Hospital. After consultation with the Japanese Pharmaceuticals and Medical Devices Agency (PMDA), the protocol of the phase I clinical trial of BNCT for cutaneous melanoma and angiosarcoma was confirmed. The clinical trial was initiated in November 2019 (ClinicalTrials.gov Identifier: NCT04293289; JapicCTI Identifier: JapicCTI-195062).^22^ The major inclusion criteria were as follows: (1) pathologically confirmed primary cutaneous malignant melanoma or angiosarcoma without lymph node or distant metastases; (2) superficial cutaneous target lesions with a maximum diameter of 15 cm or less; (3) lesions with a depth of 6 cm or less from the skin surface to the deepest part of the tumor; and (4) lesions in the head, neck, chest, or extremities. Patients who had undergone treatment previously with RT exceeding 75 Gy for the target lesion were excluded.

A simulation CT was conducted 1–2 weeks before treatment. The beam arrangement and treatment position were determined, and an approximate dose calculation was performed. According to the results of this calculation, the patient setup was reconsidered, if needed. Before and during neutron irradiation, the patients received continuous intravenous administration of SPM-011 (Borofalan [^10^B]) at a maximum dose of 500 mg/kg according to the Kyoto University method.^24^ Before the start of neutron irradiation, a blood sample was taken to measure the ^10^B concentration using inductively coupled plasma optical emission spectrometry. The treatment dose and neutron irradiation time were determined by the maximum dose to the skin, and the dose was escalated cautiously from 12 Gy-Eq using the 3 × 3 dose-escalation method during the clinical trial period. Neutron irradiation was generally completed within 30–60 min.

The treatment results were good, and the adverse events were generally not severe. Although further details cannot be reported at present because the clinical trial is ongoing, a part of the treatment methods and the authors' initial experience with BNCT for angiosarcoma patients are available elsewhere.^23^

Scalp angiosarcomas are treated with multimodal treatments, including wide excision, chemotherapy, and radiotherapy. The treatments are highly invasive, adverse events are severe, and the treatment outcomes are poor. If patients undergo surgery, wide excision with a large margin from the cutaneous lesion is required, even for small tumors. Despite highly invasive surgery, the tumor tends to recur early. Conventional radiotherapy takes a long time (6–7 weeks), which can often result in severe and extensive grade 4 dermatitis, and the patient must accept permanent alopecia around the tumor region of the scalp. In this respect, BNCT for cutaneous lesions is an effective and promising treatment with a lower risk of severe adverse events and a shorter treatment period.

#### Recurrent high-grade meningioma (WHO grade 2, 3) (jRCT 20511190044)^5^

This phase II randomized clinical trial at Osaka Medical and Pharmaceutical University [Principle Investigator (PI), Prof. Miyatake] was initiated in 2019 and was completed in August 2021.

#### Primary MG

This clinical trial was recently adopted at the University of Tsukuba as a “strategic promotion program for bridging research” sponsored by the Japan Agency for Medical Research and Development (AMED) (PI, Prof. Sakurai). They have undertaken a preclinical study and will start a phase I clinical trial in 2023.

## Conclusions

The current status of the Japanese BNCT is summarized in this article. However, further clinical trials are warranted to expand the clinical indications of BNCT for various diseases.
